# Evaluating the Efficacy, Safety, and Tolerability of Combination Therapy of Dapagliflozin and Linagliptin Over Dapagliflozin and Vildagliptin in Patients With Type 2 Diabetes Mellitus Inadequately Controlled With Metformin

**DOI:** 10.7759/cureus.58115

**Published:** 2024-04-12

**Authors:** Mala Dharmalingam, Surendra Kumar Sharma, Ved Prakash, Animesh Maiti, Ritesh Kumar, Laxminarayanappa Sreenivasa S Murthy, Balamurugan Ramanathan, Sanjiv Maheshwari, Sunil Naik Kethavath, Dhananjay Ogale, Prajapati Vipul Kumar Bachubhai, Ashutosh B Sonawane, Vaishal Shah, Manjula Suresh, Sisir Chakraborty, Krishna Kumar Manjunath

**Affiliations:** 1 Endocrinology and Diabetes, Bangalore Endocrinology and Diabetes Research Centre, Bengaluru, IND; 2 Diabetes and Endocrinology, Thyroid and Endocrine Centre, Jaipur, IND; 3 Endocrinology, Indira Gandhi Institute of Medical Sciences, Patna, IND; 4 Medicine, Kolkata Medical College, Kolkata, IND; 5 Endocrinology, Diabetes and Metabolism, Institute of Medical Sciences, Banaras Hindu University, Varanasi, IND; 6 Medicine, Life Care Hospital and Research Centre, Bengaluru, IND; 7 Diabetes and Endocrinology, Kovai Diabetes Specialty Centre, Coimbatore, IND; 8 Medicine, Jawaharlal Nehru Medical College, Ajmer, IND; 9 Medicine, Government Medical College, Srikakulam, IND; 10 Medicine, Byramjee Jeejeebhoy Medical College, Pune, IND; 11 Internal Medicine, GCS Medical College, Ahmedabad, IND; 12 Medicine, Vakratunda Hospital, Nashik, IND; 13 Medicine, Lifeline Multispeciality Hospital, Mumbai, IND; 14 Medical Services, Micro Labs Limited, Bengaluru, IND; 15 Medicine, Sagore Dutta Hospital, Kolkata, IND

**Keywords:** type 2 diabetes mellitus, glycemic control, vildagliptin, metformin, linagliptin, dapagliflozin

## Abstract

Background

Type 2 diabetes mellitus (T2DM) patients commonly undergo metformin monotherapy. This study aims to compare the efficacy, safety, and tolerability of combination therapy of dapagliflozin plus linagliptin versus dapagliflozin plus vildagliptin as add-on therapy in T2DM patients inadequately controlled on metformin.

Methodology

This was an 18-week, multicenter, randomized, double-blind, active-controlled, parallel-group, phase III clinical study. About 236 participants were randomly assigned to receive either a fixed-dose combination of dapagliflozin 10 mg plus linagliptin 5 mg tablets or a fixed-dose combination of dapagliflozin 10 mg plus vildagliptin SR 100 mg tablets added to metformin monotherapy. The primary outcome was the mean change in hemoglobin A1c (HbA1c) from baseline to the end of week 16. The key secondary endpoints were mean change in postprandial blood glucose (PPBG), fasting blood glucose (FBG), body weight, and the proportion of participants achieving HbA1c less than 7.0%.

Results

The dapagliflozin/linagliptin combination therapy showed a more significant change in HbA1c from baseline to the end of 16 weeks (mean reduction: -1.59% vs. -1.25%) compared to dapagliflozin/vildagliptin (p < 0.0001). Additionally, compared to the dapagliflozin/vildagliptin group, the dapagliflozin/linagliptin group demonstrated a significant reduction in both PPBG (mean reduction: -59.99 mg/dL vs. -55.34 mg/dL) and FPG (mean reduction: -32.91 mg/dL vs. -26.78 mg/dL). A total of 18 adverse events were reported in 17 (7.20%) participants, all of which were mild and resolved completely. There were no serious adverse events.

Conclusions

Compared to dapagliflozin and vildagliptin combination therapy, dapagliflozin and linagliptin fixed-dose combination provided clinically significant improvements in glycemic control. Because of its effectiveness, safety, and tolerability, the fixed-dose combination of dapagliflozin and linagliptin was a better option for treating T2DM patients who had previously only received metformin monotherapy.

## Introduction

According to the 2021 International Diabetes Federation report, 74.2 million adults have diabetes worldwide, with India the country with the second-highest number globally. It is projected that 124.9 million Indians between the ages of 20 and 79 years will have diabetes by 2045 [1]. Although it is well-known that maintaining good glycemic control is the best way to avoid long-term complications from diabetes, 69% of diabetic patients in India fail to reach the desired glycated hemoglobin (HbA1c) level [2]. This occurs due to inadequate glycemic control with monotherapy where combination therapy needs to be started immediately [3]. Guidelines from the American Diabetes Association, the European Association for the Study of Diabetes, and the Japanese Diabetes Society also recommend the initiation of combination therapy among patients for whom one anti-diabetic agent fails to adequately control blood glucose levels [4-6]. Several studies have shown that dipeptidyl peptidase inhibitors (DPP-4i) and/or sodium-glucose cotransporter type 2 inhibitors (SGLT2i) are two pharmacological strategies that can be added to metformin as dual therapies or combined as triple therapies [7-10].

DPP-4i, oral incretin-based therapy, is being used more frequently to treat type 2 diabetes mellitus (T2DM) as an add-on or substitute for other glucose-lowering medications, particularly sulphonylureas [11]. SGLT2i are the newest pharmacological class of glucose-lowering agents that target the kidney and induce glucosuria [12]. Recent studies have investigated their safety as well as efficacy [13,14]. The glucose-lowering actions of SGLT2i and DPP4i are mediated by distinct but complementary pathways. In the treatment of T2DM patients, a combination of SGLT2i and DPP-4i may be beneficial when one pharmacological class fails to achieve the desired HbA1c level when taken alone or even in addition to metformin [10,15,16].

Additionally, some studies on the combination therapy of SGLT2i and DPP-4i, such as dapagliflozin and saxagliptin [17], canagliflozin and teneligliptin [18], empagliflozin and linagliptin [19], and dapagliflozin and sitagliptin [20], showed that this combination therapy was well tolerated and effective to treat T2DM [2]. In T2DM patients, the fixed-dose combination (FDC) was more effective than either component alone [21]. Additionally, data indicated that in T2DM patients for whom metformin monotherapy failed to achieve adequate glycemic control, combination therapy with DPP-4i and SGLT2i significantly lowered HbA1c [19,21,22].

However, no phase III trial has previously compared the effects of linagliptin and the SGLT2i dapagliflozin on patients with other molecules of the same class in a double-blind, parallel-group setting. Hence, this study was conducted to compare the efficacy, safety, and tolerability of combination therapy of dapagliflozin plus linagliptin versus dapagliflozin plus vildagliptin as add-on therapy in T2DM patients who were poorly controlled with metformin.

## Materials and methods

**Figure 1:**
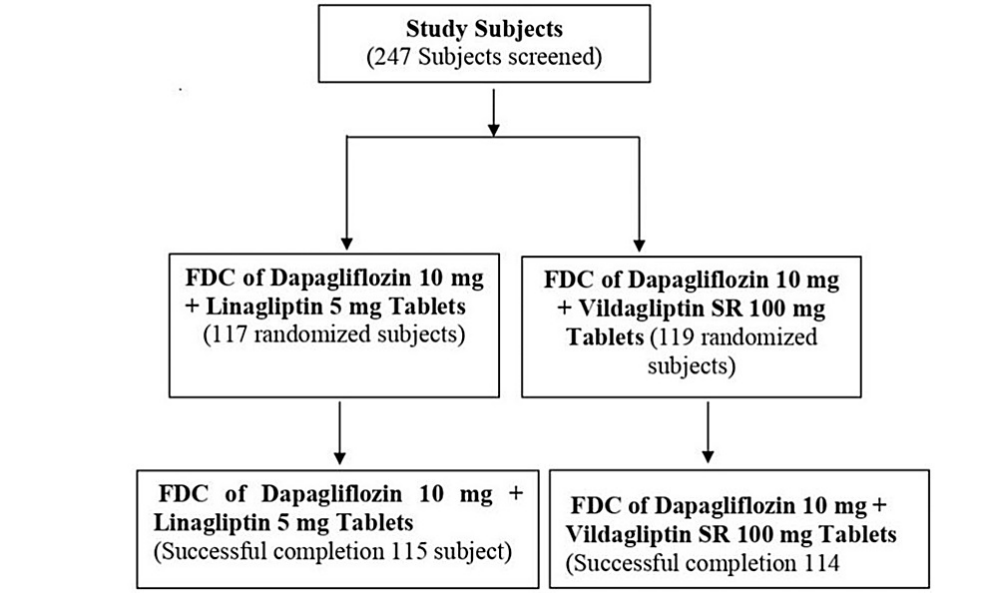
Disposition of subjects. FDC = fixed-dose combination

This randomized, double-blind, active-controlled, parallel-group, multicenter, phase III trial was conducted to compare the efficacy, safety, and tolerability of combination therapy of dapagliflozin 10 mg plus linagliptin 5 mg tablets versus dapagliflozin 10 mg plus vildagliptin SR 100 mg tablets in patients with T2DM. This study was conducted from September 2022 to March 2023 in India at 14 centers. This study enrolled participants aged ≥18 to ≤65 years of both genders with a diagnosis of T2DM, those with body mass index (BMI) less than or equal to 45.0 kg/m^2^, those with inadequate glycemic control at screening who received a stable dose of metformin of greater than 1,500 mg/day as monotherapy for at least eight weeks before screening, and those were willing to participate in this study. Exclusion criteria included women of childbearing potential if pregnant at the screening visit, breastfeeding women, those not in agreement to use adequate birth control methods to prevent pregnancy throughout the study, patients with fasting plasma glucose (FPG) >270 mg/dL at screening, and patients with other comorbid conditions such as severe hepatic impairment, chronic renal failure, thromboembolic disorders, cardiovascular disease, malignancy, immunocompromised state, or other diabetes-related complications. The disposition of patients is shown in Figure 1.

The patients were randomized in a 1:1 ratio to receive dapagliflozin 10 mg plus linagliptin 5 mg tablets (treatment A) or dapagliflozin 10 mg plus vildagliptin SR 100 mg tablets (treatment B) once daily in the morning after breakfast for 16 weeks. Study visits were scheduled at screening, at baseline (randomization), and 4, 8, 12, 16, and 18 weeks post-randomization. Telephonic visits were performed at week 4 (visit 2), week 12 (visit 4), and week 18 (visit 6).

The primary efficacy endpoint was the mean change in HbA1c from baseline to the end of week 16. The key secondary endpoints were mean change in postprandial blood glucose (PPBG), fasting blood glucose (FBG), body weight, and the proportion of participants achieving HbA1c less than 7.0% from baseline to week 16. Safety assessments were evaluated based on the findings of physical examination, 12-lead electrocardiography, laboratory parameters, reported adverse events (AEs), hypoglycemic episodes during the study, number of patients requiring rescue medications, and tolerability assessment by the investigator after treatment based on a four-point scale at the end of the study (week 18).

Ethical considerations

The clinical trial protocol was approved by the Clinical Trials Registry - India (CTRI/2022/07/044179) and various institutional review boards or independent ethics committees at the participating centers. The study was designed and monitored by ethical principles of Good Clinical Practice as defined by the International Council for Harmonization, Declaration of Helsinki, and New Drugs and Clinical Trials Rules, 2019. All participants provided written informed consent.

Statistical analysis

Descriptive statistics were used to describe the two treatment group demographics by reporting the mean and standard deviation or median as appropriate for continuous variables and numbers and percentages for binary and categorical variables. The mean change from baseline to the end of the study in both treatment groups was deduced using the paired t-test for normal data and the Wilcoxon signed-rank test for skewed data. For comparison between the two treatment groups for a continuous variable, two-sample t-tests were used if the data followed a normal distribution and the Mann-Whitney U test if the data was not normally distributed. Pearson’s chi-square test/Fisher’s exact test captured categorical or nominal variables to determine whether or not two categorical or nominal variables were likely to be related.

## Results

A total of 247 participants were screened and 236 were randomized by the SAS computer-generated randomization program. In total, 117 participants were randomized/enrolled in the FDC of dapagliflozin 10 mg plus linagliptin 5 mg tablets (treatment A) arm, and 119 participants randomized/enrolled in the FDC of dapagliflozin 10 mg plus vildagliptin SR 100 mg tablets (treatment B) arm. The demographic and baseline characteristics of the participants are shown in Table 1, with no statistically significant difference observed between both groups.

**Table 1:** Demographics and baseline characteristics. FDC = fixed-dose combination; SD = standard deviation; BMI = body mass index; PPBG = postprandial blood glucose; FBG =  fasting blood glucose

	FDC of dapagliflozin 10 mg + linagliptin 5 mg tablets	FDC of dapagliflozin 10 mg + vildagliptin SR 100 mg tablets
N	117	119
Male, n (%)	57 (48.72)	65 (54.62)
Female, n (%)	60 (51.28)	54 (45.38)
Age (years), mean (SD)	50.87 (9.58)	50.92 (9.37)
Height (cm), mean (SD)	162.40 (9.12)	162.75 (9.14)
Body weight (kg), mean (SD)	67.21 (10.65)	68.16 (10.41)
BMI, mean (SD)	25.54 (3.93)	25.74 (3.64)
HbA1c (%)	9.29	9.08
PPBG (mg/dL)	230.13	234.23
FBG (mg/dL)	166.74	166.48

**Figure 2:**
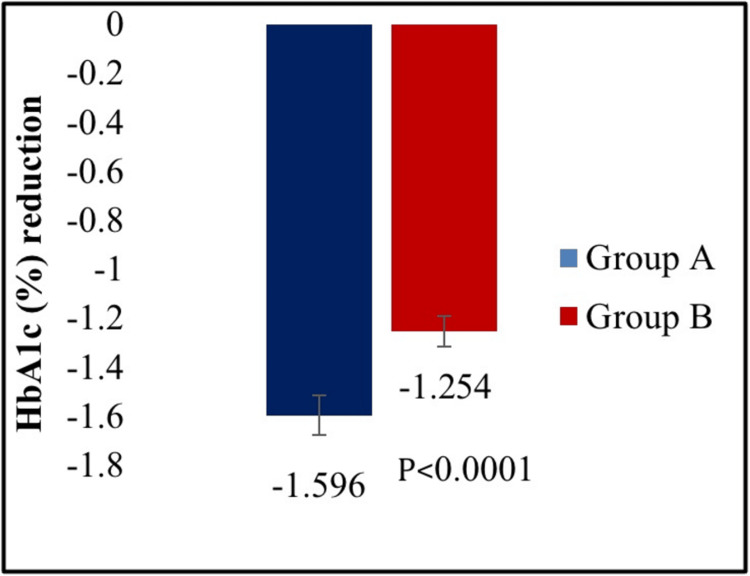
Mean reduction in hemoglobin A1c test (HbA1c) from baseline at the end of week 16.

The efficacy of the FDC of dapagliflozin 10 mg plus linagliptin 5 mg tablets was compared with the FDC of dapagliflozin 10 mg plus vildagliptin SR 100 mg tablets. There was a statistically significant reduction in HbA1c from baseline to week 16 in the treatment A group compared to the treatment B group (p < 0.0001) (Figure 2).

**Figure 3:**
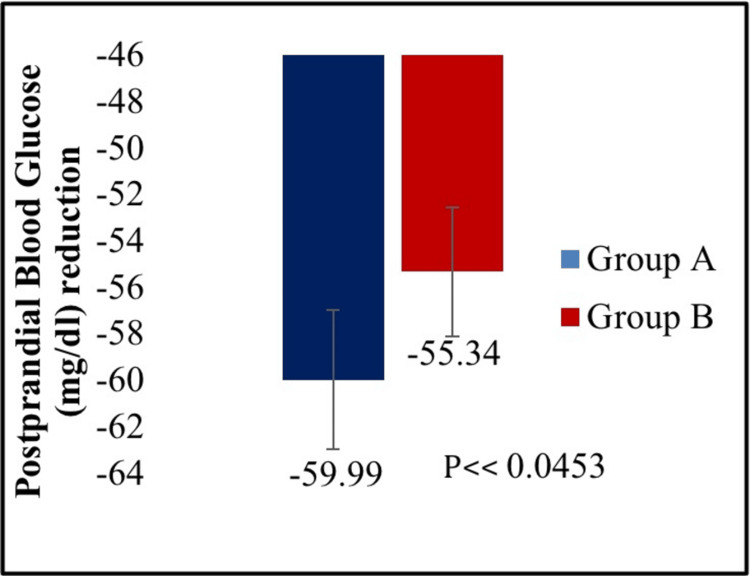
Mean reduction in postprandial blood glucose (PPBG) from baseline to week 16.

**Figure 4:**
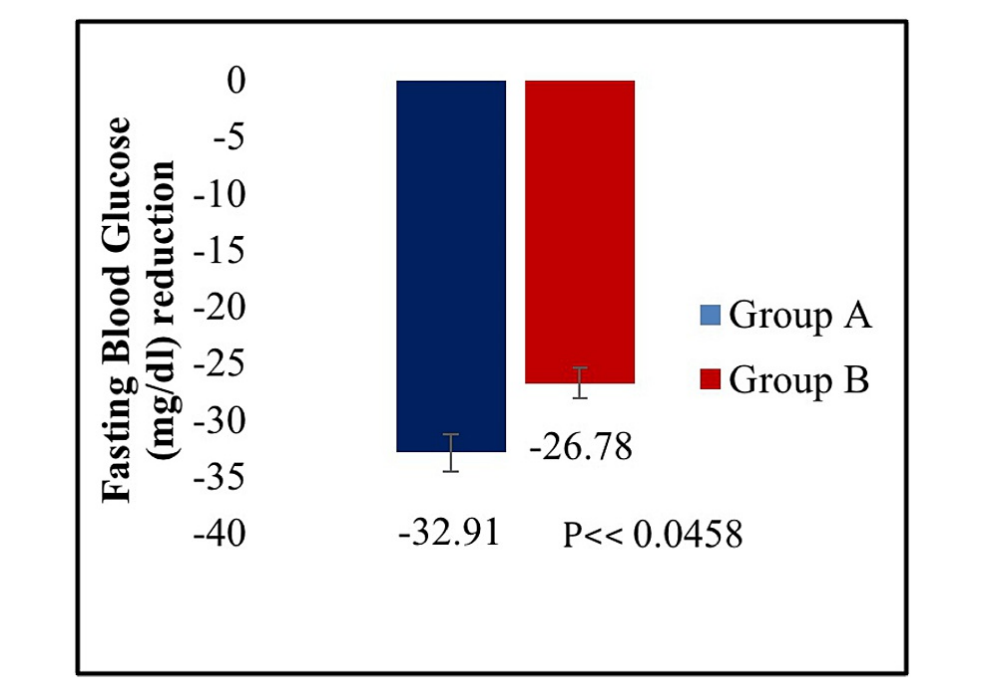
Mean reduction in fasting blood glucose (FBG) from baseline to week 16.

**Figure 5:**
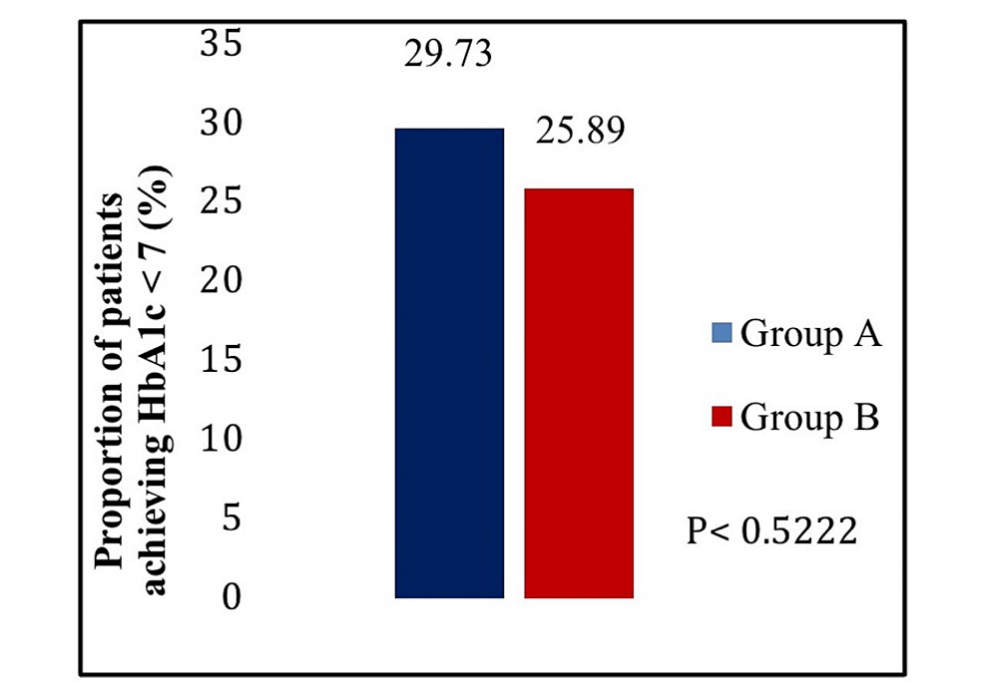
Proportion of participants achieving hemoglobin A1c test (HbA1c) less than 7.0% from baseline to week 16.

**Figure 6:**
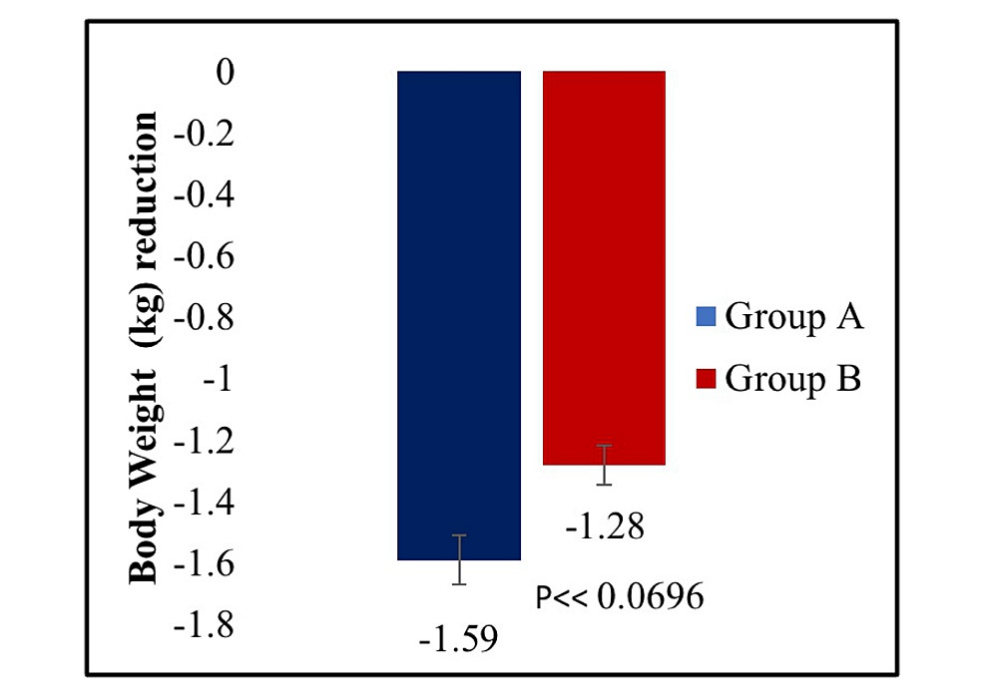
Mean reduction in body weight from baseline to week 16.

The mean reduction in PPBG was significantly greater in the treatment A group compared to the treatment B group (p < 0.0453) (Figure 3). Further, there was a statistically significant reduction in FBG in the treatment A group compared to the treatment B group from baseline to week 16 (p < 0.0458) (Figure 4). In the case of the proportion of participants achieving HbA1c less than 7.0% from baseline to week 16, no statistically significant difference was observed between the two treatment groups (p < 0.5222) (Figure 5). In addition, the change in body weight from baseline to the end of the study was not statistically significant between treatment A and treatment B groups (p < 0.0696) (Figure 6).

Safety and tolerability

The AEs reported during the trial included common cold, constipation, urinary tract infection, fever, vomiting, and headache. There were no serious AEs or deaths during the study. A total of 18 AEs were reported in 17 (7.20%) participants and all resolved completely. Of the 18 AEs, nine AEs each were noted in both treatment groups.

**Figure 7:**
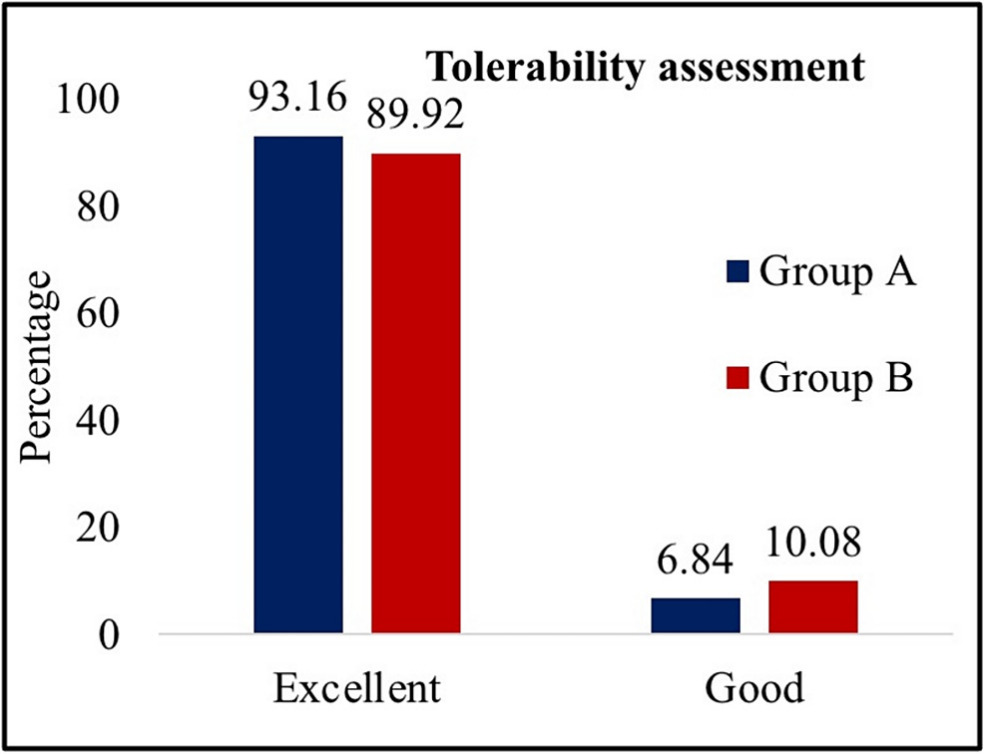
Tolerability assessment at week 18.

Tolerability assessments were done by the investigator after treatment based on a four-point scale at the end of the study, as shown in Figure 7.

Both groups tolerated the study medication throughout the 18 weeks. There were no statistically significant differences in laboratory parameters, such as electrolytes, liver function test, kidney function test, serum chemistry, lipid profile, or thyroid profile test, in the treatment groups.

## Discussion

This double-blind, active-controlled, parallel-group study is the first to compare combination therapy of dapagliflozin 10 mg plus linagliptin 5 mg tablets with dapagliflozin 10 mg plus vildagliptin SR 100 mg tablets in Indian patients with T2DM. A statistically significant reduction was observed in HbA1c (p < 0.0001), PPBG (p < 0.0453), and FBG (p < 0.0458) from baseline to week 16 in dapagliflozin plus linagliptin combination therapy group compared to dapagliflozin plus vildagliptin group. However, there was no significant difference between the two treatment groups in the proportion of participants achieving HbA1c less than 7.0% (p < 0.5222) and the change in body weight (p < 0.0696) from baseline to week 16. Thus, the results provide evidence for dapagliflozin and linagliptin FDC providing greater glycemic control than dapagliflozin and vildagliptin FDC in T2DM patients poorly controlled with metformin therapy.

The findings for HbA1c (mean reduction of 1.59% in 16 weeks) were consistent with findings from earlier trials using dapagliflozin and linagliptin combination therapy [23], indicating the clinical relevance of the research findings. In comparison to our study, the former showed a lower reduction in HbA1c (1.28% in 16 weeks). Moreover, there was also a significant reduction in FPG and PPBG with dapagliflozin and linagliptin combination therapy group which further strengthened our study findings.

In addition, compared to linagliptin, vildagliptin showed improved mean reduction in HbA1c, FPG, and PPBG, as reported by Tang et al. [24]. However, linagliptin was superior in terms of the effects on renal and hepatic function, followed by significant improvement in low-density lipoprotein levels than vildagliptin irrespective of glycemic control [25]. The addition of linagliptin with dapagliflozin further improved metabolic control without raising glucagon levels. The conflicting effects of SGLT2i and DPP-4i on glucagon levels, FPG, and PPBG may be the reason for such occurrence. While the latter raises glucagon levels while concurrently lowering FPG and PPG, the former lowers glucagon levels and either increases or does not affect FPG [26]. Several studies also supported that the combination of DPP4i and SGLT2i showed a significant reduction in HbA1c, FPG, PPBG, and body weight [18-20]. Nevertheless, this was the only study that compared these combinations and highlighted the superiority of FDC of dapagliflozin and linagliptin in patients poorly controlled with metformin monotherapy.

Overall, the safety profile of both dapagliflozin/linagliptin and dapagliflozin/vildagliptin combinations was consistent with that of the individual drug components, with a similar proportion of mild AEs noted in both groups [24]. Dapagliflozin plus linagliptin combination was generally well tolerated which was inconsistent with the findings of Jain et al. [23]. Along with better glycemic control and a similar safety profile, linagliptin with no hepatic-renal effects further enhanced that the dapagliflozin/linagliptin combination was a compelling and effective therapeutic option compared to dapagliflozin/vildagliptin combination for patients with inadequately controlled T2DM who have previously received metformin monotherapy, as supported by all of the previously mentioned findings.

Although this study was conducted only in Indian patients, it was conducted in a double-blind, active-controlled, parallel-group, phase III trial setting and included patients from multiple centers across India where the study results were supported by data from other international trials. The study captured the clinical context in which patients with poorly controlled T2DM are treated with a second-line combination therapy consisting of SGLT2i and DPP-4i. Moreover, throughout the 16-week study period, there were high completion rates (90%) and statistical power for both the primary and secondary endpoints. On the contrary, there were no significant differences in both body weight reduction and reduction in the proportion of participants achieving HbA1c of less than 7.0%. Hence, it should be confirmed with a large sample size and a longer study duration. This emphasizes the necessity to find out more about these unique qualities and explore the study parameters in different races with a wider population in the future.

## Conclusions

The comparison of dapagliflozin and vildagliptin combination therapy with dapagliflozin and linagliptin FDC provides clinically significant improvements, as evidenced by the decrease in HbA1c, FPG, PPBG values, and non-inferior in reducing body weight. Because of its effectiveness, safety, and tolerability, the FDC of dapagliflozin and linagliptin was a better option for treating T2DM patients who had previously only received metformin monotherapy. Further evidence-based studies should be performed to improve patient care and treatment plans. Hence, this combination therapy is an effective treatment procedure for T2DM patients.
